# Profile of Multidrug-Resistant Bacteria in Intensive Care Units of a Maternal and Child Hospital in Rio de Janeiro, Brazil

**DOI:** 10.3390/antibiotics14111090

**Published:** 2025-10-30

**Authors:** Lucas Meneses de Oliveira Villar, Natalie Del-Vecchio Lages Costa, Danielle Bonotto Cabral Reis, Adriana Teixeira Reis, Leticia Linhares Braga, Fabíola Cristina de Oliveira Kegele, Maria da Conceição Borges Lopes, Maria Francisca da Silva Neta Soares, Maria Elisabeth Lopes Moreira, Natália Chantal Magalhães da Silva, Leonardo Henrique Ferreira Gomes, Letícia da Cunha Guida

**Affiliations:** 1 Comissão de Controle de Infecção Hospitalar (CCIH), Instituto Nacional da Saúde da Mulher, da Criança e do Adolescente Fernandes Figueira– Fundação Oswaldo Cruz (IFF-FIOCRUZ), Rio de Janeiro 22250-020, Brazil; 2 Departamento de Enfermagem Médico Cirúrgica, Escola de Enfermagem Alfredo Pinto, Universidade Federal do Estado do Rio de Janeiro (DEMC/EEAP/UNIRIO), Rio de Janeiro 22290-180, Brazil; 3 Departamento de Patologia Clínica, Instituto Nacional da Saúde da Mulher, da Criança e do Adolescente Fernandes Figueira—Fundação Oswaldo Cruz (IFF-FIOCRUZ), Rio de Janeiro 22250-020, Brazil; 4 Unidade de Pesquisa Clínica, Instituto Nacional da Saúde da Mulher, da Criança e do Adolescente Fernandes Figueira—Fundação Oswaldo Cruz (IFF-FIOCRUZ), Rio de Janeiro 22250-020, Brazil

**Keywords:** bacterial infections, pediatric intensive care unit, neonatal intensive care unit, antimicrobial resistance, multidrug resistance, coagulase-negative staphylococci

## Abstract

**Background/Objectives:** Epidemiological surveillance of healthcare-associated infections (HAIs) and multidrug-resistant (MDR) bacteria is a key responsibility of hospital infection control committees (HICC). Active surveillance swabs facilitate the early detection of colonized patients; helping to prevent MDR pathogen transmission in intensive care units. This study aimed to describe antimicrobial resistance profiles of bacterial isolates from clinical samples in neonatal and pediatric intensive care units. **Methods:** A retrospective cross-sectional study was conducted at a maternal and child hospital in Rio de Janeiro, Brazil including patients aged 0–18 years admitted to neonatal (NICU), surgical (SICU), and pediatric (PICU) intensive care units between January and December 2023. A total of 286 positive cultures were analyzed from different sample types including blood, urine, tracheal aspirates, cerebrospinal fluid (CSF), and catheter tips as well as screening swabs (nasal and rectal) for colonization surveillance. Bacterial isolates were identified and tested for antimicrobial susceptibility following BrCAST (Brazilian Committee on Antimicrobial Susceptibility Testing) guidelines. **Results:** Of the 286 cultures, 146 (51%) originated from the NICU, 70 (24%) from the SICU, and 70 (24%) from the PICU. Coagulase-negative staphylococci (CoNS) predominated in blood cultures, especially among neonates, while MRSA was found in all nasal swabs. Among the Gram-negative bacteria; *Klebsiella pneumoniae* and *Pseudomonas aeruginosa* were the most frequent isolates, with 30–50% resistant to third-generation cephalosporins or carbapenems. ESBL-producing organisms were found in 40% of rectal swabs. **Conclusions:** The predominance of CoNS in neonatal ICUs and high resistance rates among Gram-negative bacteria highlight the urgent need for continuous microbiological surveillance and antimicrobial stewardship in vulnerable pediatric populations.

## 1. Introduction

Healthcare-associated infections (HAIs)—referred to in Brazil as “infections related to healthcare assistance” (IRAS)—are infections acquired during patient care in hospitals and other healthcare facilities [1,2]. These infections are a significant global public health problem, affecting thousands of patients annually and contributing substantially to patient morbidity, mortality, and healthcare costs [3,4]. Much modern medical and surgical care is now delivered in non-acute or ambulatory settings, blurring the traditional boundaries of where HAIs arise [5,6].

It is important to distinguish between colonization and clinical infection. Colonization denotes the presence of a pathogen on or in the patient without causing disease or an immune response [7]. In contrast, infection implies a microbe’s invasion of host tissues with associated inflammation and clinical signs (for example, fever, erythema, or purulent discharge). In intensive care units—and especially in neonatal and pediatric ICUs where children often require invasive procedures and prolonged care—colonization frequently precedes symptomatic infection. Of note, the hands of healthcare personnel are a well-recognized vector for transmission: contaminated hands are a primary route by which organisms spread from patient to patient during routine care [8].

Hospital infection control programs prevent and control HAIs and multidrug-resistant organisms (MDROs) [9]. Infection control committees (ICCs) in healthcare institutions systematically surveil HAIs and MDROs including collecting, analyzing, and interpreting epidemiological data. Based on these data, ICCs design and implement targeted prevention strategies. For example, active surveillance cultures (screening swabs) may identify asymptomatically colonized patients early [10]. Studies have shown that routine screening and isolating MRSA-colonized patients can reduce the transmission rates in high-risk settings. However, the value of universal screening remains debated, and the indiscriminate use of surveillance cultures has not been universally recommended due to resource and cost concerns. In Brazil, data on MDRO colonization in pediatric, neonatal, and surgical (a subdivision dedicated to neonatal surgical care) ICUs are limited, underscoring the need to define local pathogen profiles [11,12]. Reported MDROs in Brazilian ICU patients include coagulase-negative staphylococci and *Staphylococcus aureus* among the Gram-positives, and *Klebsiella pneumoniae*, *Pseudomonas aeruginosa*, *Acinetobacter baumannii*, and *Escherichia coli* among the Gram-negatives [13].

Understanding the local epidemiology of HAIs is essential to guide infection prevention and empirical therapy. Surveillance data inform appropriate contact precautions and antimicrobial stewardship and help clinicians select empiric antibiotics that cover the most likely resistant pathogens. Indeed, as hospitals strive to improve quality and reduce costs, robust infection control programs are increasingly viewed as critical to preventing infections and curbing the spread of MDROs [14,15].

In this context, the present study aimed to describe the antimicrobial resistance profiles of bacterial isolates obtained from neonatal, neonatal surgical, and pediatric intensive care units at a maternal and child hospital in Rio de Janeiro, Brazil, to identify the most prevalent multidrug-resistant organisms (MDROs), and provide data to support targeted infection control interventions and optimize empirical antibiotic therapy for critically ill pediatric patients.

## 2. Results

A total of 286 positive cultures were obtained: 146 from the clinical-profile neonatal ICU, 70 from the surgical-profile neonatal ICU, and 70 from the pediatric ICU. The distribution of pathogens by sample type is shown in Table 1, Table 2 and Table 3. Detailed antimicrobial susceptibility profiles, including specific resistance patterns to β-lactams, aminoglycosides, and carbapenems, are available in the Supplementary Materials (Tables S1–S3).

In the pediatric ICU, the most prevalent pathogens identified in blood cultures were *Staphylococcus epidermidis*, with seven positive cultures (63.3%), followed by *Staphylococcus hominis*, with two (18.2%), and *Staphylococcus haemolyticus* and *Burkholderia cepacia* complex, with one each (9.1%) (Table 1).

Regarding urine cultures with growth after 48 h of hospitalization, *Klebsiella pneumoniae* was the most frequently identified organism, with two positive cultures (66.7%), followed by *Stenotrophomonas maltophilia* with one positive culture (33.3%) (Table 1).

Among the tracheal aspirates, the most common pathogens were *Pseudomonas aeruginosa* and *Stenotrophomonas maltophilia*, each with seven positive cultures (30.4%), followed by *Burkholderia cepacia* complex with four (17.4%), and *Serratia marcescens*, *Klebsiella oxytoca*, *Acinetobacter baumannii* complex, *Staphylococcus aureus*, and *Enterobacter cloacae* were each isolated in one culture (4.3%) (Table 1).

Regarding cerebrospinal fluid (CSF) cultures, only one case of growth yielded *Staphylococcus epidermidis* (100%) (Table 1).

In terms of colonization, methicillin-resistant *Staphylococcus aureus* (MRSA) was identified via nasal swabs in seven cases (100%). Extended-spectrum β-lactamase (ESBL)-producing organisms were detected in seven rectal swabs, while *Pseudomonas aeruginosa* was isolated in six rectal swab cultures including strains resistant to cephalosporins and carbapenems. Additionally, one rectal swab grew *Stenotrophomonas maltophilia*, which was sensitive to sulfamethoxazole/trimethoprim (Table S1).

Regarding the neonatal intensive care unit (NICU), the most prevalent microorganism identified in positive blood cultures (BC) was *Staphylococcus haemolyticus*, with eight (34.8%) positive results, followed by *Staphylococcus epidermidis* with six (26.1%) positive cultures, *Staphylococcus aureus* with three (13.0%), *Enterobacter cloacae* with two (8.7%), and *Enterococcus faecalis*, *Pseudomonas aeruginosa*, *Serratia marcescens*, and *Staphylococcus warneri*, each with one (4.3%) positive BC (Table 2).

The most frequently identified microorganisms for urine cultures (UCs) with growth after 48 h were *Enterobacter cloacae*, *Enterococcus faecalis*, and *Klebsiella pneumoniae*, each with two (20%) positive cultures. These were followed by *Serratia marcescens*, *Pseudomonas aeruginosa*, *Acinetobacter baumannii* complex, and *Staphylococcus aureus*, each with one (10%) positive culture (Table 2).

In tracheal aspirates, *Pseudomonas aeruginosa* and *Staphylococcus aureus* were each identified in two (28.6%) positive cultures. *Acinetobacter baumannii* complex, *Klebsiella pneumoniae*, and *Stenotrophomonas maltophilia* were each identified in one (14.3%) positive culture (Table 2).

Eight positive results were observed for the following pathogens in cerebrospinal fluid (CSF) cultures: Staphylococcus warneri, Staphylococcus aureus, Staphylococcus lugdunensis, Staphylococcus hominis, Klebsiella pneumoniae, and Staphylococcus haemolyticus, each representing one (16.7%) positive culture (Table 2).

In nasal swabs (NS), *Staphylococcus aureus* resistant to methicillin (MRSA) was identified in all 74 (100%) positive cultures, confirming widespread nasal colonization by this pathogen among the patients admitted to intensive care units (Table 2 and Table S2).

Regarding the surveillance cultures, in rectal swabs (RS), *Pseudomonas aeruginosa* was the most frequently isolated microorganism, with eleven (26.8%) positive results—seven (17.1%) multidrug-sensitive isolates and four (9.7%) resistant to cephalosporins and carbapenems. Extended-spectrum β-lactamase (ESBL)-producing organisms were identified in 30 (73.2%) rectal swabs, indicating a high rate of colonization by resistant Enterobacteriaceae (Table 2 and Table S2).

Concerning colonization, the most prevalent organisms were MRSA, ESBL-producing bacteria, and *Pseudomonas aeruginosa*, with 74, 30, and 11 positive cultures, respectively (Table 2).

Regarding the surgical neonatal intensive care unit (SICU), analysis of positive blood cultures (BC) revealed that the most prevalent microorganisms were *Staphylococcus epidermidis*, with four (23.5%) positive cultures, followed by *Staphylococcus hominis* with three (17.6%). *Klebsiella pneumoniae*, *Staphylococcus haemolyticus*, and *Staphylococcus aureus* each accounted for two (11.8%) positive cultures. *Pseudomonas aeruginosa*, *Staphylococcus saprophyticus*, *Enterobacter cloacae*, and *Klebsiella aerogenes* were each identified in one (5.9%) positive blood culture (Table 3 and Table S3).

About the central catheter tip cultures (CCTCs), only one positive culture was observed for *Enterobacter cloacae*, and no positive cerebrospinal fluid (CSF) cultures were detected (Table 3).

Regarding colonization, the most prevalent microorganisms were ESBL-producing organisms, MRSA, and *Pseudomonas aeruginosa*, with 17, 12, and 7 positive cultures, respectively (Table 3 and Table S3).

## 3. Discussion

The present study provides a comprehensive overview of multidrug-resistant organisms (MDROs) isolated from neonatal, surgical, and pediatric intensive care units (NICU, SICU, and PICU) at a maternal and child hospital in Rio de Janeiro, Brazil. Coagulase-negative staphylococci (CoNS), including *S. epidermidis*, *S. hominis*, *S. haemolyticus*, and *S. saprophyticus*, were the most prevalent microorganisms isolated from the blood cultures, particularly among neonates with central venous catheters. This emphasizes the clinical relevance of CoNS as opportunistic pathogens in intensive care settings, where invasive devices are frequently used.

Consistent with previous national reports, CoNS accounted for the majority of bloodstream infections in our cohort, corroborating data from Brazilian NICUs where they are responsible for 40–60% of late-onset sepsis cases [16]. Historically considered non-pathogenic commensals, CoNS are now recognized for their ability to form biofilms and cause serious infections, particularly in immunocompromised patients and those with indwelling medical devices [17,18,19,20]. Their biofilm-forming capacity enhances virulence by facilitating tissue invasion, adherence to surfaces, and immune evasion [21,22]. Consequently, CoNS have become leading causes of nosocomial infections in NICUs, associated with a spectrum of conditions from conjunctivitis to late-onset sepsis [21,23].

*Klebsiella pneumoniae* emerged as the most frequently isolated Gram-negative pathogen from central venous catheter cultures. The 73% prevalence of ESBL-producing Enterobacteriaceae in rectal swabs from the NICU was notably higher than the rates reported in similar pediatric units (30–50%) [1,14,24,25], underscoring the critical need for local epidemiological surveillance. The emergence and spread of carbapenemase-producing *Enterobacteriaceae*, particularly KPC-producing *K. pneumoniae*, represent a major public health concern in Brazilian healthcare facilities [24]. Monitoring local susceptibility patterns using accessible laboratory methods is essential for the early identification of carbapenemase-producing strains and the timely implementation of infection control measures [25].

*Pseudomonas aeruginosa* also appeared as a clinically important pathogen, especially in patients with prolonged hospitalization, tracheostomies, or mechanical ventilation. This finding aligns with global data highlighting *Pseudomonas* as a major pathogen in hospital-acquired pneumonia [6,26]. Its intrinsic and acquired resistance to multiple antimicrobial classes facilitates persistence in healthcare environments and the colonization of medical equipment [26,27,28]. Early detection of *P. aeruginosa* colonization is clinically relevant, as MDRO acquisition frequently occurs within 5–7 days after admission [12], reinforcing the importance of strict admission screening and ongoing microbiological surveillance in pediatric ICUs.

Regarding *Staphylococcus aureus*, although most isolates remain susceptible to vancomycin [27], the detection of methicillin-resistant *S. aureus* (MRSA) in our cohort underscores persistent epidemiological challenges. The universal nasal colonization by MRSA observed in this study contrasts with previously reported rates of 40–70% [28], suggesting strong local transmission dynamics. MRSA is associated with significant morbidity and mortality, emphasizing the need for continuous surveillance and targeted infection prevention strategies [29,30].

*Stenotrophomonas maltophilia* was also identified as an emerging pathogen, particularly in patients exposed to prolonged hospitalization, catheter use, or tracheostomy. Its multidrug resistance—including resistance to β-lactams, quinolones, and aminoglycosides—combined with its environmental persistence, increases the risk of contamination and nosocomial transmission [30].

An important observation was that most cerebrospinal fluid (CSF) infections occurred in neonates with implanted ventricular shunt systems such as ventriculoperitoneal (VP) or external ventricular drains (EVD). CoNS predominated in these cases, consistent with prior reports highlighting the importance of strict aseptic protocols and device surveillance [1,31].

Overall, our findings provide valuable insight into the local epidemiology of MDROs across neonatal and pediatric intensive care settings. The predominance of CoNS and the high colonization rates of MRSA, ESBL-producing Enterobacteriaceae, and *Pseudomonas aeruginosa* emphasize the urgent need for multidisciplinary infection control programs, strict hand hygiene, catheter care protocols, and robust antimicrobial stewardship. Although limited by its retrospective and single-center design, this study reinforces the importance of continuous surveillance and evidence-based interventions to optimize empirical antibiotic therapy in critically ill neonatal and pediatric populations.

This study is limited by its retrospective, single-center design and reliance on infection control committee data, which may affect its generalizability. Nevertheless, our findings support targeted infection control interventions and provide evidence to optimize empirical antibiotic therapy in critically ill pediatric populations.

## 4. Materials and Methods

### 4.1. Study Design and Population

This was a cross-sectional, retrospective, and descriptive study conducted in neonatal, neonatal surgical care, and pediatric intensive care units (ICUs) at a public reference hospital for child and adolescent health in Rio de Janeiro, Brazil. The study population consisted of hospitalized children aged 0 to 18 years who were admitted to the pediatric and neonatal ICUs between January 2023 and December 2023.

### 4.2. Inclusion and Exclusion Criteria

Patients were eligible if they were admitted to either the neonatal or pediatric ICU for more than 48 h and had a previously negative surveillance swab. Patients with known colonization at admission and/or a positive surveillance swab within the first 48 h of hospitalization were excluded.

### 4.3. Specimen Collection and Categorization

Clinical specimens were obtained to identify multidrug-resistant (MDR) bacteria. For analytical purposes, specimens were categorized as “secretions”, which included samples from surgical wounds, drains, and operative sites. In addition, swab samples used to assess the antimicrobial resistance profiles of Gram-positive and Gram-negative bacteria were collected from multiple anatomical sites including anal, axillary, nasal, oral, oropharyngeal, ostial, bone, rectal, ulcer, and surgical wound sites.

### 4.4. Antimicrobial Susceptibility Testing

Antimicrobial susceptibility testing and interpretation followed the guidelines established by BrCAST in 2022 and 2023 [32]. Note: BrCAST is the Brazilian Committee on Antimicrobial Susceptibility Testing, which follows the EUCAST criteria and is the standard officially recommended in Brazil by the Ministry of Health.

### 4.5. Data Collection and Setting

Data were extracted retrospectively from the infection control committee (ICC) database. The neonatal ICU comprised two distinct units: a clinical care unit with fourteen intensive care beds, eight conventional intermediate care beds (UCINCo), and a surgical unit with six beds. The pediatric ICU had a general profile and included six beds. Data collection was based on medical records, and all information was handled in accordance with the ethical standards.

### 4.6. Data Analysis

A descriptive statistical analysis was conducted to determine the prevalence and distribution of multidrug-resistant (MDR) bacterial colonization among hospitalized children. Data were entered and organized using Microsoft Excel^®^ (version 2010, Microsoft Corporation, Redmond, WA, USA). Frequencies and proportions were calculated, and the results were presented as absolute numbers and percentages. The findings were summarized in tables for clarity and subsequently compared with data reported in the existing literature to contextualize and interpret the results.

### 4.7. Ethical Considerations

The study was conducted in compliance with Resolution No. 466/2012 of the Brazilian National Health Council (CNS), which regulates research involving human subjects. The study protocol was submitted to the Research Ethics Committee (CEP) via Plataforma Brasil and received formal approval under protocol number 79553224.7.0000.5269 on 16 May 2024. A waiver of informed consent was requested, as the study relied on ICC data without direct patient contact or exposure to identifiable personal information. All research team members signed a confidentiality agreement to ensure privacy and data protection.

## 5. Conclusions

Understanding the antimicrobial resistance profiles of hospital-acquired pathogens is essential for establishing an effective microbiological surveillance system and guiding empirical therapy in healthcare-associated infections (HAIs). In our study, coagulase-negative staphylococci (CoNS) were the most prevalent pathogens in neonatal and pediatric ICUs, and multidrug-resistant organisms such as MRSA, ESBL-producing Enterobacteriaceae, and *Pseudomonas aeruginosa* were also frequently identified. Patients in intensive care settings are particularly vulnerable to late-onset infections due to the frequent use of invasive medical devices and broad-spectrum antibiotics, factors that contribute to the emergence of antimicrobial resistance. Collaboration between infection control committees and multidisciplinary healthcare teams is fundamental to prevent infections and reduce the spread of resistant pathogens. Strengthening microbiological surveillance, promoting rational antibiotic use, and implementing antimicrobial stewardship programs remain critical measures to improve outcomes for critically ill pediatric patients. This study is limited by its retrospective, single-center design and reliance on existing infection control committee data, which may affect the generalizability of the findings; nevertheless, it provides valuable insights to guide local infection prevention strategies and optimize empirical therapy.

## Figures and Tables

**Table 1:** Distribution of pathogens—pediatric intensive care unit (PICU).

Sample Type	Pathogen	N	%
Blood culture	Coagulase-negative staphylococci (CoNS) *	10	90.9
	*Burkholderia cepacia* complex	1	10.1
Urine culture (UC)	*Klebsiella pneumoniae*	2	66.7
	*Stenotrophomonas maltophilia*	1	33.3
Tracheal aspirate	*Pseudomonas aeruginosa*	7	30.4
	*Stenotrophomonas maltophilia*	7	30.4
	*B. cepacia complex*	4	17.4
	*Enterobacter cloacae*	1	4.3
	*S. aureus*	1	4.3
	*A. baumannii* complex	1	4.3
	*Klebsiella oxytoca*	1	4.3
	*Serratia marcescens*	1	4.3
Cerebrospinal fluid (CSF)	*S. epidermidis*	1	100
Nasal swab	*MRSA* **	7	100
Retal swab	*Pseudomonas aeruginosa*	6	42.8
	ESBL ***	7	50
	*Stenotrophomonas maltophilia*	1	0.8

* Coagulase-negative staphylococci (CoNS)—in blood culture—*S. epidermidis*, *S. hominis*, and *S. haemolyticus*. ** MRSA—methicillin-resistant *Staphylococcus aureus*. *** ESBL—extended-spectrum beta-lactamase-producing organisms.

**Table 2:** Distribution of pathogens—neonatal intensive care unit (NICU).

Sample Type	Pathogen	N	%
Blood culture	Coagulase-negative staphylococci (CoNS) *	14	65
	*S. aureus*	3	13
	*S. warneri*	1	4.3
	*Enterobacter cloacae*	2	8.7
	*Enterococcus faecalis*	1	4.3
	*P. aeruginosa*	1	4.3
	*Serratia marcescens*	1	4.3
Urine culture (UC)	*Enterobacter cloacae*	2	20
	*Enterococcus faecalis*	2	20
	*K. pneumoniae*	2	20
	*Serratia marcescens*	1	10
	*P. aeruginosa*	1	10
	*A. baumannii*	1	10
	*S. aureus*	1	10
Tracheal aspirate	*P. aeruginosa*	2	28.6
	*S. aureus*	2	28.6
	*A. baumannii*	1	14.3
	*K. pneumoniae*	1	14.3
	*Stenotrophomonas maltophilia*	1	14.3
Cerebrospinal fluid (CSF)	Coagulase-negative staphylococci (CoNS) *	2	33.4
	*S. warneri*	1	16.7
	*S. aureus*	1	16.7
	*S. lugdunensis*	1	16.7
	*K. pneumoniae*	1	16.7
Nasal swab	MRSA **	74	100
Retal swab	*Pseudomonas aeruginosa*	11	26.8
	ESBL ***	30	73.2

* Coagulase-negative staphylococci (CoNS)—on blood culture—*S. epidermidis* and *S. haemolyticus*. In CFS: *S. hominis* and *S. haemolyticus*. ** MRSA—methicillin-resistant *Staphylococcus aureus*. *** ESBL—extended-spectrum beta-lactamase-producing organisms.

**Table 3:** Distribution of pathogens—surgical neonatal intensive care unit (SICU).

Sample Type	Pathogen	N	%
Blood culture	Coagulase-negative staphylococci (CoNS) *	10	55.8
	*K. pneumoniae*	2	11.8
	*S. aureus*	2	11.8
	*P. aeruginosa*	1	5.9
	*Enterobacter cloacae*	1	5.9
	*Klebsiella aerogenes*	1	5.9
Central catheter tip cultures (CCTCs)	*Enterobacter cloacae*	1	100
Nasal swab	MRSA **	17	100
Retal swab	*Pseudomonas aeruginosa*	7	36.8
	ESBL ***	12	63.2

* Coagulase-negative staphylococci (CoNS)—in blood culture—*S. epidermidis*, *S. haemolyticus*, and *S. saprophyticus*. In CFS: *S. hominis* and *S. haemolyticus*. ** MRSA—methicillin-resistant *Staphylococcus aureus*. *** ESBL—extended-spectrum beta-lactamase-producing organisms.

## Data Availability

The data presented in this study are available on request from the corresponding author. The data are not publicly available due to institutional restrictions and patient confidentiality.

## Supplementary Materials

The following supporting information can be downloaded at: https://www.mdpi.com/article/10.3390/antibiotics14111090/s1, Table S1. Detailed antimicrobial susceptibility profiles—NICU; Table S2. Detailed antimicrobial susceptibility profiles—SICU; Table S3. Detailed antimicrobial susceptibility profiles—PICU.

## Abbreviations

The following abbreviations are used in this manuscript:

CCTC	Central catheter tip culture
CoNS	Coagulase-negative *Staphylococcus*
CSF	Cerebrospinal fluid
CVC	Central venous catheter
EVD	External ventricular drain
ESBL	Extended-spectrum beta-lactamase
HAIs	Healthcare-associated infections
HICC	Hospital infection control committee
ICC	Infection control committee
ICU	Intensive care unit
IRAS	Infections related to healthcare assistance
KPC	*Klebsiella pneumoniae Carbapenemase*
MDR	Multidrug-resistant
MDROs	Multidrug-resistant organisms
MRSA	Methicillin-resistant *Staphylococcus aureus*
NICU	Neonatal intensive care unit
PICU	Pediatric intensive care unit
SICU	Surgical intensive care unit
UC	Urine culture
VP	Ventriculoperitoneal
