# Supplementary material for: Profile of Multidrug-Resistant Bacteria in Intensive Care Units of a Maternal and Child Hospital in Rio de Janeiro, Brazil
Source: Antibiotics (Basel). 2025 Oct 30;14(11):1090. doi: 10.3390/antibiotics14111090 (PMC12649471; doi:10.3390/antibiotics14111090)
Supplement: Supplementary file 1 [file antibiotics-14-01090-s001.zip › antibiotics-3869934-supplementary.pdf]

Supplementary Material

The following tables provide detailed antimicrobial susceptibility data for all isolates identified in the Neonatal (NICU), Surgical (SICU), and Pediatric (PICU) Intensive Care Units. These tables include full antibiotic sensitivity profiles for each pathogen.

Table S1. Detailed antimicrobial susceptibility profiles – NICU

**Sample Type**	**Isolated Microorganism**	**Antibiotic Resistance Profile**	**N (%)**
**Blood culture**	*S. epidermidis**	Sensitive to vancomycin;	6 (60%)
	*S. hominis**	Sensitive to vancomycin;	2 (20%)
	*S. haemolyticus**	Sensitive to vancomycin;	1 (10%)
	Complexo B. cepacia	1 not tested, as BrCAST does not recommend it;	1 (10%)
**Urine culture (UC)**	*K. pneumoniae*	Resistant to gentamicin	1 (33.3)
		Sensitive to sulfamethoxazole/trimethoprim	1 (33.3)
	Stenotrophomonas maltophilia	Sensitive to sulfamethoxazole/trimethoprim	1 (33.3)
**Tracheal aspirate**	*P. aeruginosa*	Multidrug-sensitive	3 (12.9)
		Resistant to cephalosporins;	2 (8.6)
		Resistant to cephalosporins and carbapenems	1 (4.9)
	Stenotrophomonas maltophilia	Sensitive to sulfamethoxazole/trimethoprim	7 (30.4)
	B. cepacia Complex	Not tested, as not recommended by BrCAST	4 (17.4)
	Enterobacter cloacae	Multidrug-sensitive	1 (4.3)
	S. aureus	Sensitive to oxacillin and vancomycin	1 (4.3)
	A. baumannii Complex	Multidrug-sensitive	1 (4.3)
	Klebsiella oxytoca	Resistant to cephalosporins	1 (4.3)
	Serratia marcescens	Resistant to 3rd-generation cephalosporin	1 (4.3)
**Cerebrospinal**	*S. epidermidis*	Sensitive to vancomycin	1 (100)

**Fluid (CSF)**			
**Rectal swab**	*P. aeruginosa*	Multidrug-sensitive;	4 (23.5)
		Resistant to cephalosporins ;	4 (23.5)
		Resistant to cephalosporins and carbapenems	1 (5.8)
	*ESBL***		7 (41.1)
	Stenotrophomonas maltophilia	Sensível a sulfametoxazol/trimetoprim	1 (5,8)
**Nasal swab**	*MRSA****	Resistant to methicillin, sensitive to vancomycin	7 (100)

* Coagulase-negative staphylococci (CoNS)- In Blood culture - *S. epidermidis and S. hominis. In CSF- S. haemolyticus.*
** MRSA - Methicillin-resistant *Staphylococcus aureus.*
***ESBL - Extended-spectrum beta-lactamase-producing organisms.

Table S2. Detailed antimicrobial susceptibility profiles – SICU

**Sample Type**	**Isolated Microorganism**	**Antibiotic Resistance Profile**	**N (%)**
**Blood culture**	*S. haemolyticus**	Sensitive to vancomycin	8 (34.8)
	*S. epidermidis**	Sensitive to vancomycin	6 (26,2)
	S. aureus	Sensitive to oxacillin and vancomycin	3 (13.0)
	S. warneri	Sensitive to vancomycin and oxacillin	1 (4.3)
	Enterobacter cloacae	Multidrug-sensitive	2 (8.7)
**Urine culture (UC)**	*Enterobacter cloacae*	Sensitive to cephalosporins, carbapenems, amicacin, and gentamicin	2 (20)
	Enterococcus faecalis	Sensitive to vancomycin and ampicillin	2 (20)
	K. pneumoniae	Sensitive to cephalosporins, carbapenems, and amicacin, resistant to gentamicin;	1 (10)

		Multidrug-sensitive	1 (10)
	Serratia marcescens	Multidrug-sensitive	1 (10)
	P. aeruginosa	Multidrug-sensitive	1 (10)
	A. baumannii	Sensitive to gentamicin, resistant to amikacin and carbapenems	1 (10)
	S. aureus	Sensitive to oxacillin and vancomycin	1 (10)
**Tracheal aspirate**	*P. aeruginosa*	Resistant to cephalosporins and carbapenems	2 (28.6)
	S. aureus	2 Sensitive to oxacillin and vancomycin	2 (28.6)
	A. baumannii	1 sensitive to oxacillin and vancomycin	1 (14.3)
	K. pneumoniae	1 Resistant to gentamicin	1 (14.3)
	Stenotrophomonas maltophilia	Sensitive to sulfamethoxazole/trimethoprim	1 (14.3)
**Cerebrospinal Fluid (CSF)**	*S. warneri*	Sensitive to oxacillin and vancomycin	1 (16.7)
	S. aureus	Sensitive to oxacillin and vancomycin	1 (16.7)
	S. lugdunensis	Sensitive to oxacillin and vancomycin	1 (16.7)
	*S. hominis**	Sensitive to oxacillin and vancomycin	1 (16.7)
	K. pneumoniae	multidrug-sensitive	1 (16.7)
	*S. haemolyticus**	Sensitive to vancomycin	1 (16.7)
**Rectal swab**	*P. aeruginosa*	7 multidrug-sensitive	7 (17.1)
		Resistant to cephalosporins and carbapenems	4 (9.7)
	*ESBL***		30 (73.2)
**Nasal swab**	*MRSA****	Resistant to methicillin, sensitive to vancomycin	74 (100)

* Coagulase-negative staphylococci (CoNS)- In Blood culture - *S. epidermidis and S. hominis. In CSF- S. hominis and S. haemolyticus.*

** MRSA - Methicillin-resistant *Staphylococcus aureus.*

***ESBL - Extended-spectrum beta-lactamase-producing organisms.

Table S3. Detailed antimicrobial susceptibility profiles – PICU

Sample Type	Isolated Microorganism	Antibiotic Resistance Profile	N (%)
**Blood culture**	*S. epidermidis**	Sensitive to vancomycin	4 (23.5)
	S. hominis	Sensitive to vancomycin	3 (17.6)
	K. pneumoniae	ESBL, resistant to gentamicin, sensitive to carbapenems and amikacin	1 (5.9)
		Multisensitive	1 (5.9)
	*S. haemolyticus**	Sensitive to vancomycin	2 (11.8)
	S. aureus	Multisensitive	1 (5.9)
		MRSA sensitive to vancomycin	1 (5.9)
	P. aeruginosa	Multisensitive	1 (5.9)
	*S. saprophyticus**	Sensitive to vancomycin	1 (5.9)
	Enterobacter cloacae	Multisensitive	1 (5.9)
	Klebsiella aerogenes	Multisensitive	1 (5.9)
**Urine culture (UC)**	*Enterobacter cloacae*	Sensitive to cefepime, amikacin, and gentamicin, not tested for carbapenems.	1 (100)
**Rectal swab**	*P. aeruginosa*	Resistant to cephalosporins and carbapenems;	1 (5,2)
		Sensitive to carbapenems and resistant to cephalosporins;	2 (10.4)
		Resistant to cephalosporins and imipenem, sensitive to meropenem;	1 (5.2)
		3 Multisensitive	3 (15.6)
	*ESBL***		30 (73.2)
**Nasal swab**	*MRSA****	Resistant to methicillin, sensitive to vancomycin	17 (100)

* Coagulase-negative staphylococci (CoNS)- In Blood culture - *S. epidermidis*, *S. haemolyticus* and *S. saprophyticus.*

** MRSA - Methicillin-resistant *Staphylococcus aureus.*

***ESBL - Extended-spectrum beta-lactamase-producing organisms.
